# A principled and cosmopolitan neuroethics: considerations for international relevance

**DOI:** 10.1186/1747-5341-9-1

**Published:** 2014-01-03

**Authors:** John R Shook, James Giordano

**Affiliations:** 1 Philosophy Department and Graduate School of Education, University at Buffalo, Buffalo, NY, USA; 2 Neuroethics Studies Program, Pellegrino Center for Clinical Bioethics, Georgetown University Medical Center, 4000 Reservoir Road, Bldg D Rm 238, Washington, DC 20057, USA; 3 Human Science Center, Ludwig-Maximilians Universität, Munich, GER, Germany

**Keywords:** Neuroscience, Prescriptive neuroethics, Principled neuroethics, Cultural pluralism, Meta-ethics, Cosmopolitanism, Medical ethics

## Abstract

Neuroethics applies cognitive neuroscience for prescribing alterations to conceptions of self and society, and for prescriptively judging the ethical applications of neurotechnologies. Plentiful normative premises are available to ground such prescriptivity, however prescriptive neuroethics may remain fragmented by social conventions, cultural ideologies, and ethical theories. Herein we offer that an objectively principled neuroethics for international relevance requires a new meta-ethics: understanding how morality works, and how humans manage and improve morality, as objectively based on the brain and social sciences. This new meta-ethics will simultaneously equip neuroethics for evaluating and revising older cultural ideologies and ethical theories, and direct neuroethics towards scientifically valid views of encultured humans intelligently managing moralities. Bypassing absolutism, cultural essentialisms, and unrealistic ethical philosophies, neuroethics arrives at a small set of principles about proper human flourishing that are more culturally inclusive and cosmopolitan in spirit. This cosmopolitanism in turn suggests augmentations to traditional medical ethics in the form of four principled guidelines for international consideration: empowerment, non-obsolescence, self-creativity, and citizenship.

## International neuroethics

The scientific foundations of neuroethics are structured upon advances in the brain and behavioral sciences, and in the novel technologies that allow access, evaluation, and manipulation of the brain and its functions, inclusive of the amalgam of conscious processes, cognitions, and emotions that contribute to the ‘mind’ and/or the ‘self’. The ever-broadening use of neuroscience and neurotechnology arouses scrutiny of longstanding ‘common sense’ and philosophical concepts of the relation of brain to mind, and compels inquiry to the validity and value of these ideas – and their implications – in the scientific, medical and socio-cultural realms.

It is in this critical light that the philosophical foundations for neuroethics are also gradually, yet steadily organizing. (Examples of broadly philosophical treatments of neuroethics include Levy [1] and the work of Racine [2]). These foundations are based in part upon extant constructs of science, mind, self, and social relations, and yet, we opine that there is an increased need for their re-examination and perhaps reconstruction in light of new information from the brain sciences, to update epistemological, anthropological, and ethical norms. Better understanding of how those normative sources have functioned for humanity to date – especially because they can now be openly scrutinized – can then be leveraged in formulating concepts, constructs, and constraints regarding the ways that neuroscientific research could and should be conducted and applied in medicine to evoke effect(s) within cultures and the social sphere. Clearly, neuroethics will be an essential part of any such view [3-5]. Prescriptions for what ought to be done about these implications soon follow. Thus, neuroethics will be inescapably prescriptive, and justifications for those prescriptions will rely on normative premises. Normative premises are abundantly available: social, moral, and legal norms abound from all directions and every culture. Neuroethics might remain prescriptively splintered by such normative diversity and conventionality. Therefore, we ask if neuroethics – as a philosophical field – can define and settle on core norms to take a unified principled stance? If it can, where will those normative premises be found, which ethical principles for neuroethics would be wise, and what policy and legal regulations would follow from such ethical principles?

We assert that pondering a unified principled stance for neuroethics is not an idle speculative venture. The field of neuroethics is confronted with urgent international questions of how to deal with brain research and the uses of novel neurotechnologies originating in many countries and quickly crossing borders, whether from benevolent, commercial, or even malevolent intent. Looking globally, neuroscience and neurotechnology are no longer the province of Western nations, as shifts in scientific, technological and economic capabilities are evermore enabling non-Western countries to become viably engaged in a growing international market of neuroscience (currently estimated at greater than $150 billion annually). This shifting balance will necessitate addressing ethical, legal, and social issues incurred through the use of neuroscience and technology not only in developed nations, but in those that are developing and under-developed, as well. The worldwide discussion of neuroscience and neuroethics has swelled, and will undoubtedly continue to increase [6,7]. Calls for a global neuroethics relevant to upgrading international policies and laws are mounting accordingly [8-11]. As a field and set of practices, neuroethics should be involved in these international deliberations, because its theoretical resources allow direct examination and evaluation of the human being, and human predicament (of disease, illness, suffering and finitude) from a metaphysically and methodologically naturalistic grounding and perspective that is 1) well comported with medicine, 2) conciliatory toward human cultural diversity and 3) not incompatible with theological views. Accordingly, we further urge that neuroethics should forge philosophical foundations and theoretical ethics that are universally and objectively valid as science itself. To this end, we address the following core issues. How might neuroscientific information about putative bases of moral cognitions and actions be engaged to establish a basis for the development of ethical systems and practices that are naturalistically grounded? Can such neuroethical deliberations be guided by more than just one culture’s ethical ideals in order to guide the ways that neuroscientific research is conducted and applied on the world stage?

We affirmatively answer these questions in five stages. First, the primary modes of prescriptive neuroethics are outlined, showing how their argumentative forms admittedly fit better with social conventionality than with ethical theorizing. Second, a path for neuroethics to transcend inadequate ethical theorizing and outdated meta-ethics is cleared, a new meta-ethics for neuroethics is revealed, and hopes are posed that neuroethics can undertake ethical theorizing. Third, neuroethics is shown to be compatible with a modest type of cosmopolitan ethics that we believe will be important to a broader, more naturalistic, and culturally inclusive ethico-legal discourse. Fourth, in the spirit of cosmopolitanism, (four) principled guidelines for a more internationally capable neuroethics are proposed for consideration: Empowerment, Non-obsolescence, Self-creativity, and Citizenship. Finally, this philosophical path from ‘synapse to society’ and on to a principled international neuroethics is defended against expected objections.

### Prescriptive neuroethics

Pro Roskies [12], neuroethics has inherently (if not axiomatically) embraced two central matters: first, studying neural function to understanding how our species – and others – developed and manifest capacities for sociality and morality; and second, undertaking ethical thinking about researching and modifying neural structure and functions of cognitions, emotions, and behaviors using the techniques and technologies of neuroscience. The first mode of neuroethics explores how new knowledge about the functions of the brain may impact wider understandings of self, social relations, and culture. The second mode of neuroethics explores how such self- and socio-cultural understandings should be applied to judging the implications and potential effects of neuroscientific research and its employment in various domains of the social sphere. Pondering how new neuroscientific information about the processes of intentional volition may indicate modifications to criteria for criminal responsibility and just punishment is an example of the first mode; pondering whether convicted criminals should receive novel brain modifications to diminish their anti-social conduct is an example of the second.

Both modes have factually descriptive components [2]; both are normatively prescriptive as well. The prevalence of prescriptivity throughout neuroethics deserves more attention. The dual-aspect nature of neuroethics is generally acknowledged, but the disadvantages of bifurcating neuroethics into ‘traditions’ such a ‘neuroscience of ethics’ contrasted with an ‘ethics of neuroscience’ should also be recognized [13-15]. Distinctions can inflate into dichotomies, especially where the gravity of traditional dichotomies exert philosophical pull. The ‘is-ought’ divide can particularly sway an ethics of neuroscience towards enveloping all of the prescriptive work. On the contrary, the way that the neuroscience of ethics recommends adjustments to our conceptions of self, morality, and society necessarily involves sensitively important normative and ethical issues [16]. A non-normative and ‘purely descriptive’ neuroethics only appears feasible where some common notion of sociality or morality is appraised as unquestionably correct and made the object of research. This ‘pure’ description of ‘the way humans do things’ hides its normative prescriptivity behind a façade of unrecognized cultural conventionality. As soon as this ‘purely descriptive’ neuroethics is forced to notice how differing conceptions of sociality and morality are available for selective research, its purity is adulterated by normativity. Furthermore, any neuroethical judgment that sociality or moral responsibility needs to be re-conceived in light of fresh neuroscience exposes how this ‘descriptive’ neuroethics is already on prescriptive territory, since a specific norm of sociality or moral responsibility is getting selected for scrutiny, and some alteration to that norm is recommended for its better ‘fit’ with the current recognized facts about brain function afforded by neuroscience. Both modes of neuroethics are unavoidably prescriptive. Furthermore, the dual modes of neuroethics must be intricately connected across both descriptive and prescriptive dimensions, since novel self-conceptions must affect methods of doing ethics, which in turn will change how ethical norms are applied to proposed brain technologies that can further modify (self)-conceptions of humanity.

To avoid chaotically changing everything at once, philosophical reflection typically approaches matters piecemeal. For both modes of doing neuroethics, even the most sophisticated arguments yielding prescriptions can exemplify a basic form. For the first mode, some item of neuroscientific knowledge is premised in order to justify modifying certain socio-cultural views. Hold the science steadily in view, and recommend socio-cultural change to keep a good fit with the realities science affords. For the second mode, some view of the human being and/or socio-culture is premised in order to justify a verdict on the appropriateness of employing a neuroscientific technique or technology in research or practice. Here, hold the self-socio-cultural view steadily in view, and use those norms to evaluate neuroscientific change(s) to brain structure and functions of cognition, emotion and/or behavior. Both modes basically hold one side of the neuroscientific/self-socio-cultural formula steady, and recommend what must be done (or not done) to the other to maintain some sense of balance or coherence.

At first glance, the philosophical quest for coherence and stability sounds reasonable enough. However, abundant resistance arises from all directions to obstruct revisions to self-socio-cultural matters, or to prevent deployment of novel technologies. Prevailing cultural traditions and ideologies (including folk psychologies, common morals, religious traditions, economic and political systems, etc.) mount resistance to modifying conceptions of the human being/society/culture, especially where those conceptions have normative dimensions. Struggles over brain science that might be relevant to sensitive matters such as gender or sexuality, family bonds and roles, personhood status and autonomy (e.g., of the mentally ill or criminals) supply just a few examples. Struggles just as easily erupt over opportunities to utilize novel technologies. On most any issue, opposed positions tend to develop and harden: one camp conservatively rejects using a new technology by appealing to stable tradition, while the other camp progressively recommends a novel social structure made possible by some new technology [17]. Both camps appeal to anything useful at hand, such as moral intuitions, common social standards, cultural norms, and legal rules. Indeed, so many of these are available for recruitment by both sides that neither camp may prevail, resulting in deadlock.

Only where there is wide agreement on priorities would we expect to see somewhat easier convergence on accepting some change in views of the human being, society and culture, and the use of new technologies. Specifically, a society will more quickly and compliantly accept new life technologies when that society is already highly committed to some important goals, such as lifespan extension, mental health, or crime prevention. Where neuroethics is concerned, public justifications for using neurotechnologies to modify physiological functions and behaviors will largely take a ‘socially conventional’ form, as a society appeals to what it considers as valid and binding norms and goals. Without question, social-cultural norms can and do afford a vast amount of practical work and public benefit. In their more rigid form as legal statutes, such norms are often quite proper, and arguably necessary for social order. Prescriptive judgments coalesce into legal and policy regulations as needed.

Neuroethics must pay due attention to cultural traditions, prevailing ideologies, and social conventions. Indeed, much of neuroethics will remain beholden to those powerful sources of norms and ideals, making tacit or explicit appeals to them in the course of urging prescriptive judgments. Yet, however attractive and useful, these normative sources do not supply universally accepted principles – people disagree within societies, societies disagree with each other, and entire cultures gradually change over time. Just because a large part of a society, or much of a culture, happen(s) to prefer things a certain way does not automatically make it right, good, and/or just. What can appear to be the ‘strongest’ ethical arguments are really only locally and modestly prescriptive, and permitting majority-based social standards to speedily decide matters may actually perpetuate deep ethical disagreements rather than resolve them. If philosophical foundations of/for neuroethics remain at this socio-cultural level, argumentative stalemates will be frequent, and even where broad norms weigh in favor of one position, those norms will still be only socio-culturally relative and such positions have no wider ‘objective’ status. Prescriptive neuroethics at its best may remain philosophically fragmented, with an objectively principled neuroethics remaining out of reach. Of course, such a neuroethics would hardly be the only ‘applied’ ethics to be so fragmented – there is a growing recognition of irreducibly pluralistic bioethics in general [18-21].

Offers to rescue neuroethics (and bioethics) from this fragmented situation have been offered from those claiming that there are universally valid norms for all humanity. Theologically inspired offers rarely comport well with the scientific worldview, but even if that clash could be overcome, religious traditions tend to disagree with each other over ethics as much as cultures do. The naturalistically-minded philosophers among the theological community often appeal to preserving ‘humanity’, ‘human nature’, ‘human virtues’, and the like. Their naturalism, however, prevents this strategy from rising above conventionality as much as hoped. In this Darwinian age, such essentialist appeals can only amount to aggregating nicer humans into one set and pointing to what many of us happen to be doing well [22-24]. For example, repudiations of futurist plans of trans-humanist agendas and post-humanisms typically make claims that either amount to “what humans have been doing as morally right is a path from which no one should stray,” or “matters should they remain as they have been.” (That is why the divergent values of some future ‘post-human’ society are typically disregarded by such conservative arguments.) Promoters of trans-humanism and post-humanism are quite capable of appealing to selected ‘universal’ norms of humanity as well, but closer examination of this strategy exposes how these norms tend to be conveniently pre-selected from special phases of civilization and then ‘discerned’ within all humanity [25].

To be sure, philosophy has additional resources. Established ethical theories, such as various deontologies, utilitarianism, contractarianism, and virtue ethics, may be ways to surmount conservative-progressive stand-offs, and rise above socio-cultural conventionalism altogether. These ethical theories lay claim to some higher ‘objective’ status, but do they really tend to end controversy? Far from it; the spectacle of argumentative standoffs among ethical theories lends applied ethics its characteristic adversarial tone. Any agreeable convergence among rival ethical theories seems more like a matter of chance than design. Even those ethical theories proud of a basis in ‘reason’ do not precisely agree on how to best be rational.

Does prescriptive neuroethics have any further options beyond settling for socio-cultural fragmentation, seeking humanity’s ‘genuine’ values and virtues, or following the lead of one or another established ethical theory? As a field, neuroethics has an opportunity to transcend these alternatives. By taking the social, behavioral, and brain sciences most seriously, the first mode of neuroethics has access to knowledge about how humans cognize the world, undertake their conduct, engage in relationships, and structure and manage social and moral responsibilities. The second mode of neuroethics has the capacity to apply such knowledge for evaluating the methods used for ethically judging proposed modifications to ourselves and our societies. In short, we opine that there is nothing about how we can do morality, make ethical judgments, change moral habits or social roles, or re-design societies that is theoretically off-limits or beyond the purview of neuroethics. This burdens neuroethics with the requirement of being consistent with several sciences (bringing attendant concerns discussed in the next section), but it simultaneously loosens neuroethics from complete dependence on folk psychologies, social conventions, cultural standards, obsolete epistemologies and theories of mind, traditional philosophical and religious ethics, and outdated meta-ethics.

Has neuroethics fully realized the extent of a proper domain, and the potential capaciousness of its power? If not, neuroethics will remain weakly prescriptive, but it will obtain its value premises on loan from outside sources. Neuroethics can make appeals to intuitions, social conventions, legal statutes, and ethical theories too; indeed, these inherited argumentative habits from older versions of applied ethics (such as medical ethics) nearly exhaust the neuroethics literature to date. But we believe that a much wider field of action awaits neuroethics: the potential to be served by – and serve as – a new meta-ethics.

### A new meta-ethics for neuroethics

Meta-ethics involves clarification of any linguistic, epistemic, psychological, or even metaphysical presuppositions and commitments involved with moral thinking and practice. Ethical theories tend to append some meta-ethics to their systems since each theory relies on a characteristic view of what morality is and how morality works, views contested by rival ethical theories. Before the advent of the behavioral and brain sciences, such meta-ethical presuppositions were just that: sheer assumptions. Philosophers and theologians ‘found’ them grounded in all sorts of places, such as folk intuitions, grammars, linguistic definitions, ‘common sense’ morals, socio-cultural norms, and legal regulations, along with whatever the ‘best’ sciences or theologies of the day said about free will, human nature, natural law, speculative metaphysics, or divine commands. Over the centuries, typical pronouncements of meta-ethical principles have really amounted to little more than personality traits, linguistic habits, folk psychology concepts, comfortable moral intuitions, race/class/gender prejudices, theological dogmas, armchair speculations, and so forth.

Ethical theories and meta-ethics have long mapped out morality and moral concepts in the absence of adequate biological, sociological, and psychological knowledge about origins of human sociality, the human capacity for doing morality, and the ability to modify moral and social norms. We posit that a new scientific meta-ethics can gain independence from inherited intuitions, social conventions, and older ethical theorizing. Neuroethics will engage the social, behavioral, and brain sciences to erect the foundations of a new meta-ethics. Neuroethics need not be another ‘applied ethics’ beholden to outdated meta-ethics or ethical theories; nor will neuroethics be imperiously told (by any postmodernist meta-ethics, for example) that bioethics cannot attain any measure of objectivity, or be cowed by an analytic meta-ethics into abandoning empirical ethics as a fallaciously naturalistic project [26]. For neuroethics, neuroscientific understandings of the subject matter, namely actual human sociality and moral cognition, take priority. In a similar manner, the behavioral and cognitive sciences are supplying much-needed tests and correctives to epistemologies, theories of learning, and metaphysical notions of the body-brain-mind relationship [27,28].

Neuroethics could exemplify how to fruitfully apply a new scientific meta-ethics because it addresses and treats three matters that are crucial to any meaningful and authentic exploration of human life: namely, moral capacity, moral practice, and moral principle. What does ‘morality’ mean to neuroethics? Roughly, the naturalistic understanding of human morality takes it to be a socially sustained practice, found in all (or nearly all) cultures, in which individuals voluntarily and habitually conduct themselves in accord with understood norms promoting personal fitness for social interactions and regulating public conduct of wide social concern. People participate in a morality not only by regulating their own behavior in social relationships, but also by assisting in the needed enforcements of moral norms, and by teaching moral norms and the means of enforcement to those who need moral education. The universality of this social technology of morality indicates its significant and longstanding utility for social groups small and large (especially when supplemented by the far older norms of kinship and the much younger norms of law) [13,29].

Let us sketch neuroethics’ approach to moral capacity, moral practice, and moral principle in that order. First, utilizing knowledge from the biological, cognitive and social sciences, neuroethics applies understandings of neural substrates and mechanisms of cognition to investigate how humans have the capacity to be social and moral [3,13]. Any theories involving mistaken presumptions about how sociality works, how we must think about morality, and the cognitive resources available for managing society or being moral, will be disproven and then suitably revised or speedily eliminated. Ideologies and philosophies having a concern for actual human morality means they can be held accountable by scientific information about human cognition and sociality. Theoretical recommendations about people being moral and becoming more moral must make at least four kinds of presumptions about (1) how people are already doing morality, in some specified sense of what it means to be ‘moral’; (2) the cognitive/emotive processes that people undertake when trying to be moral; (3) how certain changes to these processes are possible; and (4) how some of these changes can result in a person’s conduct becoming more moral. Theories making these presumptions can hence be discredited in any of four ways: (1*) a theory’s specified sense of ‘morality’ may not resemble how humans generally do morality; (2*) a theory’s view of the cognitive/emotive processes involved with doing morality may be inaccurate or entirely mistaken; (3*) a theory may be proposing modifications to processes of doing morality that are not in fact possible; or (4*) a theory’s view that possible modifications to moral processes are effective for doing morality better cannot in fact be that effective. Social ideologies and ethical philosophies are not immune from evaluation and criticism from the behavioral and brain sciences. Ethical theories that can be adapted in light of scientific knowledge will enjoy a deserved advantage [30].

Second, from this sound(er) basis in reliable theorizing about sociality and morality, neuroethics can expand its inquiry into any and all social and moral practices, carefully evaluating them for their consistency with brain realities, and recommending modifications where indicated. Expectations that people should be doing things a certain way should align with the ways that (their) brains can actually function. Neuroethics (like the brain and behavioral sciences generally) will be perpetually confronted by cultural ideologies, legal and political philosophies, ethical theories, meta-ethical systems, and the like, each protesting that factual brain science is largely irrelevant to the normative task of making people into who they ought to be. While neuroscience does not – and/or cannot purport – to prescribe and proscribe actions or establish ideals, it – and neuroethics – can infer and inform what, why and how neural functions, and effects can enable embodied organisms (like humans) to sense, perceive, emote, decide and act, and this is important to the establishment of norms and ethics about the ways we relate. Furthermore, guiding people’s lives implies shaping minds, so ignorance of the brain is no excuse. Any movement of social reform, for example, should partner with neuroethics in order to determine how modifications to brain structure and function (by whatever means, from inter-socially pedagogical to neurologically pharmaceutical) can affect our personal capacities, inter-personal relationships, and moral practices. More generally, neuroethics is usefully central to inquiries into the potential wider impacts of modified mind/brain capacities and practices on all other moral, social, economic, legal, political, cultural, (etc.) realms of life [3,5,13,31].

Third, proceeding from some sense of human moral cognition and action, and how adjustments to ourselves and our social practices may have wider implications, neuroethics can help formulate principled judgments about whether and how modifications to existing moral and wider social practices ought to be made. Having participated in the comprehension of moral capacities and the reformulation of sound ethical theorizing, neuroethics can proceed to an articulation and application of improved ethics to concrete problems arising in and from brain research and new neurotechnologies that are coming fast to the global stage. Again, established ethical systems will claim priority here, offering to stock neuroethics with their principles, but such principles can be freely accepted or declined as appropriate. Unlike philosophies that prefer to isolate objective morality and its supposedly rational basis from conventional ethics in its cultural settings, we reserve for neuroethics a meta-ethical stance that takes the cognitive and social sciences seriously in their investigations of the embodied human being embedded within socio-cultural environments [3,4,31]. This opportunity might first appear like a return to the option of socio-cultural conventionality, but, starting from science in fact opens the possibility for a far more objective foundation for neuroethics than the ‘objectivity’ promised by older ethical theories.

### Neuroethics and moral naturalism

Neuroethics is contributing to the project of moral naturalism that aims at scientifically understanding how humans practice sociality and morality in their cultures. Moral naturalism must not be confused with moral realism – when a moral naturalist proposes to study human morality, there is no specific code of morality intended and no commitment is made about whether one or another morality is ‘true’. Moral naturalism is hospitable to deep moral pluralism, although it is inhospitable to views of morality that contradict sound science [32-34]. This meta-ethical grounding for and of neuroethics in the brain and behavioral sciences arouses philosophical suspicions, too many to entirely forestall in this paper. Rather, we can only make a few statements about such concerns here. In our view, while neuroethics has no choice but to be naturalistic in its approach to studying sociality and morality, neuroethics is not automatically beholden to ethical naturalism, since neuroethics need not agree that all moral meanings and values, and any ethical principles adjudicating among them, entirely reduce to the status of objective facts about the natural world. Nor must neuroethics take a strictly eliminativist stance against freedom, agency, and responsibility, but need only consider scientifically acceptable versions that find responsible autonomy in learned capacities for managing individual conduct and social relations, rather than in some mythical ‘free will’ ignoring natural laws or non-existent ‘self-conscious decisions’ always instantaneously controlling actions (compatibilist theories grounded on social neuroscience are better scientific candidates, for example [2,35-37]). Neuroethics need not necessarily heed extant ethical theories’ criteria for possessing freedom or autonomy (such as “the capacity for purely rational thinking” and the like); nor need neuroethics be premised on any ‘neuro-essentialism’ positing that a conception of the human being cannot exceed our neurobiology [3,13,38].

Neuroethics is not reducible to any specific amount of science, yet science is crucial for meta-ethics and neuroethics. By ensuring scientific continuities between actual moral conduct in the natural world, inquiries into the conditions permitting such conduct, and prescriptions for modifying how people morally conduct themselves, neuroethics remains fully committed to the scientific worldview without reducing ethical philosophy to the sciences themselves. On this meta-ethical view, (neuro)scientific knowledge about human (or any other species’) morality is not incompatible with all ethical philosophizing. While ethical theorizing that relies on entirely disproven notions must be eliminated, claims that evolutionary psychology, sociology, or the cognitive sciences will eliminate morality itself (and obviate all ethics) are hasty and overblown [39]. The scientifically-based meta-ethics of neuroethics will find plenty of genuinely natural morality among humans to research, and this meta-ethics will leave room for neuroethics to engage ethical philosophy.

Some, but not all, ethical philosophies are refuted by the fact(s) that: many people are not fulfilling morality’s altruistic expectations; peoples’ moral intuitions have emotional roles set by evolution instead of cognitive ways to track moral realities; peoples’ intuitive notions of how morality works are quite mistaken; and ordinary language about morality is replete with confusions and errors. Ethical philosophies do not all agree about the cognitive or motivational capacities of ordinary morality, and they don’t all share the same degree of reliance upon what people happen to think or say about morality. Ethical philosophers typically focus on thoughtfully guiding people toward improving one or another system of morality – and the shortcomings obtained therein. For example, the discovery that people typically fulfill only minimal expectations of morality, and are sentimentally partial and partisan towards those like themselves who live in proximity, is not exactly a stunning revelation for much of philosophical ethics (or for religious ethics) that some brain scientists may have made it seem [39]. Similarly, when one or another ethical theory or meta-ethics has defined ‘morality’ or ‘moral knowledge’ in terms later discovered to be inadequate by the brain or behavioral sciences, philosophers should refrain from announcing that “morality does not exist,” and instead focus on discrediting (sources of) poor definitions of morality [40-42].

Despite centuries of misguided and mistaken ethical theorizing about the origins and foundations of morality, it has been and remains a robust part of human social life. Neuroethics can be an equally robust and perhaps better philosophical ethics. In general, philosophical ethics can handle less than ideally moral people and can avoid defining morality in entirely fictitious terms, but ethical theories cannot keep supposing that their preferred modes of ethical reasoning are immune to discoveries about actual human cognition. A scientifically based meta-ethics, and its focus starting from an understanding of moral cognition, emotion and behaviors in the human world means that ethical philosophizing can be held accountable by neuroethics, not the other way around. No philosophical ethics, not even utilitarianism or deontology, can enjoy presumptive ethical status anymore.

Neuroscience’s liberation from reliance upon notions of morality established by antiquated ethical theories, (that are absent knowledge about cognition), is only half-heartedly recognized at present. For example, the relative immaturity of neuroethics as a discipline and practice is manifested by the curious way that some neuroscientists are attempting to map correspondences between specialized cognitive/affective functions and the modes of reasoning inherent to traditional ethical theories (for overview, see [1,43]). Why just those ethical theories, rather than others? Are we forever wedded to utilitarianism and deontology (or any other lineup of extant theories that one would care to list)? Imagine if epistemological inquiries were conducted in this manner. That some brains are capable of thinking ‘deontologically’ and others in a ‘utilitarian’ manner when confronted with an artificial situation having only two possible outcomes only indicates that brains are indeed trainable in those two ways (which we knew well before brain imaging). But no amount of brain imaging would infer that those are the only two ways of moral thinking. The far more interesting kinds of information from neuroscience do not involve what we already know about what brains can do, but rather what brains could potentially do differently – and perhaps better. What will brain images look like from people who transcend the artificial utilitarian-deontological option when dealing with messier real-world situations? We should be looking at a neuroscience of the morally possible, not just the ethically necessary.

To be sure, while we are pointing a way towards developing a scientifically adequate meta-ethics, this essay does not offer a ‘correct’ ethical philosophy grounded in that meta-ethics. Even the lengthy process of weeding out disproven ethical theories (not attempted here) leaves no obvious single winner in its place – the negotiation between the brain and behavioral sciences and adequate ethical theorizing will be an on-going process for as long as new things are learned about cognition. Instead, we here propose undertaking three modest meta-ethical goals: First, grounding a new meta-ethics for neuroethics on empirical knowledge about actual people in their societies; second, questioning whether a prescriptive neuroethics must remain beholden to such things as socio-cultural norms or traditional ethical theories; and third, suggesting how a new neuroethical framework with objectively principled outcomes could be erected. This path from real people to normative prescriptions, and then on to neuroethics principles, is neither obvious nor easy, especially because outdated meta-ethical presumptions crowd the philosophical landscape. Surmounting conventional prescriptivity still appears especially daunting.

How can neuroethics go about selecting and elevating conventional prescriptions into objective principles? Since the meta-ethics of neuroethics must follow the brain and behavioral sciences in their view of morality as socio-culturally embedded, doesn’t that imply that all prescriptive judgment is forever limited to relativistic status? And if neuroethics would instead find its principles in some other source besides actual human cultures, would that search amount to a betrayal of its confessedly scientific foundations? We hold that there is a meta-ethical way past this dilemma. We resist a simplistic forced choice between many diverse social conventions or a unitary trans-cultural ethics for doing prescriptive neuroethics. Neuroethics’ moral naturalism and its reliance on the brain and behavioral sciences – especially cultural anthropology, social neuroscience, and cognitive neuroscience – cannot endorse that dichotomy.

Brains are certainly embodied, and people are thoroughly socialized and encultured beings [3,13]. So philosophical appeals to some mythical capacity for pure reason or detachment from group identity can’t work; people can do far more than robotically express one culture. People are not individuals with accidental cultural identities, nor are their identities exhausted by the folkways of some culture or another. At the same time, these encultured humans possess intelligent capacities to cognitively reflect on cultures [9]. Furthermore, most people can appreciate how they stand with respect to cultures, they can enjoy some emotional ability to empathize with others in different cultures, and they can learn from other cultures. The very fact that humans enjoy quite sophisticated cultures is the very reason why we can defensibly assert that we are not forever trapped within just one culture (or sub-culture, etc.) or another. Indeed, ethnic and cultural identities could not be constructed, deliberately managed, and carefully sustained against hegemonic and assimilationist pressures unless ethnic and cultural identity could be objects of reflective evaluation and comparison [44,45].

Enculturation is most powerful when it is least visible, but it can come into view in many ways. People can realize how other cultures are different, yet at the same time, not so different. People can re-evaluate their own culture’s habits and norms; people can revise their social structures in light of novel goals and ideals; people can combine cultural features or move to other cultures; people can respect and value people of other cultures without necessarily valuing everything about those other cultures; and people of different cultures can work on converging agreement on shared principles (although perhaps for differing reasons). In short, people can feel respectfully beholden to their own cultures even while they perceive that social norms can, or sometimes should, be modified. Humanity is a species that re-designs its moralities, just as it designs and modifies all social technologies. Modifying moralities cannot be a path towards some trans-cultural position, however, since at every stage of socio-technological development, we are still talking about thoroughly encultured humans [46-48]. But it is a path that permits recognition of the locations and limitations of any given socio-cultural convention. This human capacity helps to explain how cultural evolution happens at all. Furthermore, it turns out to be no paradox that we can travel across and partially transcend socio-cultural boundaries through our capacities for understanding the very existence and permeability of those boundaries.

Summarizing, neuroethics should participate in forging a new, objective meta-ethics based upon scientific research into human societies and their moralities. This new meta-ethics in turn grounds the needed neuroethical testing of ethical theories for adequacy, which then permits neuroethics to suggest improvements to our understandings of morality and to ethical theories, and explain why humans have the cognitive resources to reflectively modify socio-cultural inheritances. Modifying social structures such as moralities is far from easy; in the short term, domestic appeals to social convention get much practical and policy work done. All the same, methods yielding short term, local results don’t necessarily work beyond their social range of application, or their conventional premises.

### Principled and cosmopolitan neuroethics

We now come to the question of whether the evident capacity of neuroethics to be prescriptive on its own philosophical terms provides for the further ability to become objectively principled as well. Although neuroethics can and should take advantage of a new meta-ethics grounded in the brain and behavioral sciences to acquire some degree of liberation from socio-cultural conventions, cultural ideologies, and outdated ethical theories, this progress is insufficient to guarantee that neuroethics could erect an objectively principled ethical position. Understanding which conventions, ideologies, and ethical theories to avoid is hardly the same thing as discovering the one right ethical system. Neuroethics could still remain forever fractured, prescriptive only for local situations and social contexts, and valid only by being premised on group or cultural norms. Within any actual society, of course, prescriptive neuroethics can seem properly principled, as it contributes to the reflective stability of norms for that society. The larger question is whether a principled neuroethics can apply to far more than just local contexts in a piece-meal fashion. Will the philosophy and practices of neuroethics rise above social or cultural relativism? Can neuroethics provide something of objective value to the world at large?

Thus far, this essay has sought to arouse a creative tension between (A) the way that neuroethics respects how human brains are embodied, socialized and encultured; (B) the expectation that neuroethics can and will do its prescriptive work with great sensitivity to socio-cultural-historical contexts; and (C) the hope that neuroethics could approach an inter-cultural level of principled philosophical ethics. But we hold that the tension within and between these points is resolvable by the fusion of their concepts and tasks. Specifically, we think that a new meta-ethics for neuroethics is already entailed within points A and B: that is, – respect for both the power of enculturalization and the intellectual flexibility to modify cultures. People are always encultured, yet they can be thoughtful and creative individuals, who can contribute to cultural comparison and change. We believe that this position points the way toward fulfilling the hopes of point C.

As we view it, a new meta-ethics for neuroethics already contains some principled treatments of sociality and enculturalization that bridge the transition from how humans are successfully social, to ways they should continue to be social. For example, humans are properly encultured to permit opportunities for their flourishing, yet cultural essentialism is unsound. So we should be suspicious of social groups preventing individuals from changing their self-identities, dictating the identities of its members, aggressively assimilating new members, or denying their members’ efforts to learn and think about the ways of their culture and those of other cultures. Ethnocentrism is similarly unsound, so we should be suspicious of any society claiming to exemplify a ‘correct’ way of life. Along these lines, we can see why excessive cultural elitism is unsound, since no society/culture is so elite or correct that it can reasonably classify the members of other societies as sub-human or less worthy of respect or dignity. Cultures still permit people to pass moral judgments on others (that’s the point of having a morality), but individuals in other cultures are still to be viewed as worthy candidates for moral regard [49].

Following this train of thought, excessive nationalism looks unsound as well. While citizenship can be a valuable status for people, no country should presume that a person’s identity or loyalty is primarily characterized by one’s current domicile or citizenship, and people should not automatically prioritize their nation’s interests. Because the new meta-ethics of neuroethics will also remain skeptical towards any ethical theorizing that lays claim to trans-cultural or absolute status, this stance renders implausible any political theory reliant upon those sorts of foundations, such as certain kinds of political liberalism or social contractarianism grounded on a vision of ‘true’ human nature that fails to recognize and regard biology and culture [50].

A new meta-ethics will not merely describe how humans are social and moral within cultures, since it will also comprehend how ecologies are capable of providing conditions for successful human understanding and improvement of their cultures. Most relevantly, this neuroethical meta-ethics will grasp the proper cultural conditions minimally needed for people to intelligently manage, sustain and improve their moralities. Inappropriate cultural conditions are hence specifiable as well, and include: obstructing knowledge about how sociality and morality works; preventing people from intelligently questioning and creatively modifying their social structures and moralities; isolating people to keep them ignorant about other cultures; promoting ideology that one’s own culture must be uniquely correct; encouraging people to demean and demonize those in other cultures; and generally stunting the human capacity (such as it is) for empathy and cooperation with others. Cultures that foster such inappropriate conditions are not fulfilling their proper function, basically by failing to enhance intelligent human flourishing, which is the entire point of being encultured humans. The universality of the use of culture across humanity supplies the key to locating cultural norms to encourage.

Such norms obtain: respect for individuals who value their identities and are changing their self-identities; opportunities for people to acquire capacities for flourishing; protection of individuals from cultural insulation, isolation, and ignorance; denial that any society has exclusively correct norms; disdaining efforts to cast some peoples outside the circle of full humanity; and valuation of people for themselves and not merely with regard for their heritage, citizenship, or political status.

One tradition of ethical and political philosophy highly prioritizes all of these recommendations: cosmopolitanism. Humanist in its ethics, liberal in its attention to rights, and open to secular as well as religious freedom – but not oppression or aggression – in its politics, cosmopolitanism has long supported ethnic toleration, cultural pluralism, equal rights, liberal democracy, global cooperation, and international peace [51-55]. Cosmopolitanism cannot be uncritically adopted, of course. Over the course of its history, some varieties of cosmopolitanism have harbored hegemonic, essentialist, trans-cultural, or putatively absolutist principles among their foundations. Cosmopolitanism has occasionally included among its first principles unrealistic expectations about such things as a human motivation to prioritize and follow reason; a human capacity for deep empathy and equal concern for all; a willing suspension of concern for local matters to tackle distant situations; an eager altruism for supplying strangers with plentiful support at the cost of much personal wealth; an excessive tolerance for moral and cultural pluralism; an anti-pluralist hope for one hegemonic world culture; a determination to view humanity only as one community; or a drive to abolish countries in favor of a single world government. Varieties of cosmopolitanism can evidently be not only idealistically hopeful about humanity, but as unrealistic as any ethical or political philosophy could be. We opine that the naturalistic meta-ethics for/of neuroethics cannot support the aforementioned cosmopolitanisms that are reliant on these sorts of expectations.

However, we assert that a modest cosmopolitanism, compatible with typical moral performance, hospitable to people enjoying ethnic diversity and democratic self-determination, and workable with contemporary political structures such as nations, international bodies, and global accords, makes a good fit with the new meta-ethics as we have formulated [56-59]. Despite prevalent caricatures of cosmopolitanism as a way for privileged Westerners to ‘discern’ agreeable moral rules ecumenically ‘shared’ by other cultures, only to blunder into cultural misunderstandings and perpetuate colonialist stereotypes, we venture to support a more philosophically sophisticated cosmopolitan stance. We caution that neuroethics would be wise to abstain from commitments about broader issues as wealth egalitarianism, economic globalization, personal property rights, or humanity’s political solidarity [60,61]. Judging the appropriate political frameworks for realizing cosmopolitan visions, or deciding whether and when primary citizenship could be transferred from a country to a world polis is well beyond the purview of neuroethics alone. All the same, a principled, cosmopolitan neuroethics can be involved with offering recommendations for intercultural deliberation about crucial issues such as guaranteeing basic freedoms, protecting everyone from harms, promoting material and cultural opportunities for all, and preserving peoples’ capacities for self-governance.

### Four guidelines of a principled neuroethics

We have opened a reasoned path from the scientific foundations for a novel objective meta-ethics towards a principled cosmopolitan neuroethics. The next task of translating the high ideals of this cosmopolitan neuroethics into practical prescriptions about potential applications of neuroscience and neurotechnologies is not any easier. What are needed are mid-level principles to guide ethical and policy deliberations in concrete situations. Fortunately, neuroethics is hardly the first discipline to seek those sorts of principles. The heritage of medical ethics is conspicuously available in this regard.

If neuroethics is to transcend social conventionalism, the relationships between neuroethics and medical ethics are necessarily going to be complex. As a discipline, neuroethics is a sub-field of bioethics, which considers the moral implications of the life-sciences, and since study of neural systems is among the life sciences, neuroethics falls under bioethics as an academic discipline [2]. Yet, while engaging the inter-disciplinarily of bioethics, the methodology of neuroethics will need to be partially liberated from bioethics and from medical ethics in particular [2,3,13,43]. Medical ethics to date has been dominated by problems of Western medicine and ideals of Western philosophy, which are premised on normative notions of the ‘moral individual’, what counts as ‘standard health’, and concerns for the ‘autonomous patient’ (what we have coined as ‘MISHAP’). Indeed, medical ethics has had a generally conservative track-record, as befits a field trying to prevent medico-moral mishaps in these domains [62,63].

By contrast, in practical application, neuroethics is both more specific and broader than medical ethics, because neuroethics must consider how and why individuals, non-state organizations, and governments will be utilizing brain/mind modifications for pursuing the widest imaginable variety of goals from pleasure to violence, both within countries and across international borders [3,13,31,64]. As for undertaking principled ethics, neuroethics will partially transcend medical ethics, precisely because neuroethics must regard modifications to the brain/mind made for any reason within and across cultural or political boundaries, including transitions to future iterations of humans, cultures, and/or beings yet to emerge.

It must be acknowledged that medical ethics and its application of principles such as beneficence, non-maleficence, respect for autonomy and justice has been truly useful for grappling with the impacts of scientific knowledge and technologies [65,66]. These ‘mid-level’ ethical principles have made much good sense in the scientific context of medicine, and within the social contexts of Western culture, but they are not without contention [67], and in the light of neuroethical questions and dilemmas, we pose that they no longer entirely suffice. Novel neuroscientific technologies will soon expose the inherent limitations of all four principles as understood so far.

For example, respect for autonomy presumes that there is an individual who has a stable personal identity over time, but radical cognitive modifications will permit the creation of new selves. Whose autonomy has been violated when someone has re-written most of their own memories? Beneficence presumes that there are objectively identifiable goods to be pursued by health care providers, but radical modifications will be undertaken by individuals who will decide for themselves what is valuable for their own lives. Who is to judge the harms of radical cognitive modifications when undertaken by people to gain competitive advantages in the workplace? Non-maleficence presumes that there are objectively identifiable harms for health care providers to avoid, but radical modifications will be chosen by individuals who will decide for themselves what ‘harms’ are acceptable. Where is the harm in eliminating the need for sleep without side-effects? Justice presumes that there are scarce medical resources to be distributed by health care providers (or governments) in some equitable manner, but some kinds of radical modifications will be selectively funded by communities, corporations, militaries, and countries to make people more useful in assigned jobs. Where is the injustice in obtaining a radical modification in order to stay employed in a well-paying profession, or receiving radical adjustments to courage and sensitivity levels to heighten performance as a peace officer?

The tradition of Western medical ethics and the four principles mentioned here (and similar principles gone unmentioned) [68,69] are not well-designed for such future scenarios. To be perfectly fair, however, justifications for principled medical ethics have frequently appealed to the way that beneficence, autonomy, non-maleficence, and justice are widely respected by many of the world’s civilizations, ethical systems, and wisdom traditions [70-72]. It is not a coincidence that twentieth century medical ethics has overlapped a great deal with modern cosmopolitan ideals. Selected ideals of medical ethics could be revised for fulfilling cosmopolitanism to a much higher degree. Practical continuity between principled neuroethics and medical ethics has many advantages. We agree with Eric Racine’s pragmatic view that neuroethics should transformatively adapt useful bioethical work, rather than reinvent or duplicate bioethics [2]. While a new scientific meta-ethics may supersede outdated ideologies and philosophies, such meta-ethics cannot directly derive specific moral codes, so it would be impractical for a principled neuroethics to attempt a blank-slate start [3,5,13,31]. Evolutionary continuity reconciles this principlism with pragmatism (a pragmatic heuristics unable to suggest guiding principles is empty, after all), and the kind of principlism suggested here should be understood as the ethical prioritization of important moral ideals, rather than the rationalistic imposition of moral ‘axioms’ from which applied deductions must derive. This pragmatically flexible approach fully permits thoughtful balancing and adjudication among these ethical priorities when applying them to specific cases, and it encourages their perpetual testing and reconstruction in a manner consistent with the scientific meta-ethics of neuroethics.

Summing up thus far, we have argued that progress towards an objectively principled neuroethics can be made by naturalistically reconstructing ideals of medical ethics and augmenting them according to a modest cosmopolitanism. To illustrate how this pragmatic ethical evolution may proceed, we suggest four augmented guidelines for international consideration: empowerment, non-obsolescence, self-creativity, and citizenship.

Augmenting beneficence yields *empowerment*. The duty to advance the welfare of others should be extended to the duty to increase the capabilities of people to autonomously live independent and fulfilling lives. A modification would be considered to be unethical if it causes unreasonable harms to a person, makes a person more dependent on others (especially to the point of losing effective citizenship, the fourth guideline), or reduces a person’s capacity to pursue one’s own well-being.

Augmenting non-maleficence yields *non-obsolescence*: The duty to avoid unreasonably harming people should be extended to avoid the creation of obsolete people, especially ‘single-use’ people that are so irreversibly specialized by radical body/brain modifications that career and lifestyle options become too limited. A modification is unethical if it unreasonably risks producing a person with peculiar or radical ‘enhancements’ that excessively restrict future self-creativity, or if it reduces empowerment or citizenship.

Augmenting autonomy yields *self-creativity*: The right of persons to autonomously direct their lives should be extended to the right to re-create themselves for enriching their lives. Access to self-creative modifications, even to the point of making new selves, should be protected, so long as other guidelines are respected along the way. Self-creativity must not be conflated with individuality or peculiarity; people should also be allowed to re-create themselves to more closely conform to desired group standards (so long as those standards do not themselves involve loss of autonomy or violations of the other three guidelines). A modification is unethical if it contracts creativity; for example, by reducing responsible autonomy or capacity for further creativity, reducing basic capabilities for supporting one’s self, or limiting potential competencies to improve one’s standard of living and well-being.

Augmenting justice yields *citizenship*. The duty to fairly distribute scarce goods should be extended to the duty to guarantee everyone’s ability to be a free, equal, law-abiding, and participatory citizen. A modification would be unethical if it risks debilitating a person’s capacity for fulfilling the roles and responsibilities of engaged civic life, or enjoying the rights and obligations of citizenship.

While we are confident that a cosmopolitan neuroethics (indeed, a cosmopolitan bioethics in general) would be wise to include principled guidelines like these, we don’t wish to exaggerate the priority of just these four specific principles. A different formulation of cosmopolitanism and a different selection from traditions of medical ethics would result in variant sets of guidelines. The process of finding the best mutual adjustment among a new neuroscientific meta-ethics, improved ethical theorizing, and inter-cultural principles for global utility has only begun.

### Cosmopolitan neuroethics

We have claimed that neuroethics can find its philosophical foundations in much the same way that its scientific foundations are found in understanding the human brain. The objectivity of the new meta-ethics for neuroethics cannot exceed the degree of scientific objectivity involved, but there is robust objectivity available nonetheless, and that objectivity can infuse a cosmopolitan neuroethics as well. We propose fairly objective cosmopolitan principles for neuroethics out of a responsible sense of pragmatic need in light of current world affairs and with a view to recommending international protocols, conventions, and treaties [73]. Our approach seeks ethical objectivity not in trans-human rationality but in inter-human deliberations [48]. Only inter-cultural principles are sought here, not trans-cultural or absolutist norms.

We have not paradoxically acknowledged cultural and moral pluralism only to heedlessly forge ahead with imposing a universalistic ethics or unitary vision of ‘the’ good life without regard to actual human experience. We do not risk any self-contradiction by first protesting against both the traps of conventionalism and the dreams of absolutism, and then offering guidelines that should sound persuasive to many if not most contemporary cultures. Nor do we risk self-contradiction by dismissing principles grounded on essentialist and eternal visions of humanity and then resorting to an objective meta-ethics grounded in how humans commonly attempt to live best at present. Our proposal does not elevate one culture’s norms to universalist status over humanity, but rather it seeks the universal norms inherent to cultures doing their proper work for humanity. Objective ethical theorizing need not be held hostage to self-destructive cultural ideologies and outdated philosophies inconsistent with the natural facts of human cognition, sociality, and moral capacity. As we all are well aware, plenty of cultures instruct fantastical notions about mental abilities, inculcate doctrines hostile to healthy social relationships, and perpetuate power inequalities by reserving full moral autonomy only to the few. Refusing to permit their veto over an objective global ethics is entirely consistent with the cosmopolitan standpoint on proper cultural functioning and promoting human flourishing.

The modest cosmopolitanism urged here lends serious support to efforts to preserve cultural diversity and self-determination in the face of assimilation and hegemony (although it cannot aid cultural essentialism or isolationism). Our four proposed principles can help address understandable worries that radical biomedical enhancement could undermine human sociality and solidarity [74], and they can preserve moral achievements made by civilizations around the world to date, which are impressive enough that even postmodern pluralists tacitly appeal to their validity. While conservative in spirit from a certain perspective, these principles can also be viewed as liberal and even liberating. If the advocates of the most daring trans-humanist and post-humanist visions are able to admit that contemporary adherence to these cosmopolitan principles would be fairly useful for heading towards their dreams from where humanity is standing today, then we may avoid accusations of having prejudiced ethical theory against them.

Neuroethics cannot avoid its destined role for deep investigations of humanity and broad relevance to humanity’s problems and potentials. Neuroscience, neurotechnology, and a host of other scientific and technological advancements can and will change the human predicament, if not the human being. Socio-cultural forces will both affect the scope and conduct of neuroscience and technology and be affected by them in turn. Neuroethics will remain valid, viable, and of value by boldly participating in the science-based evaluation of these interacting dynamics and by helping to flexibly guide those dynamics on an international, pluralized world stage.

## Competing interests

The authors declare that they have no competing interests.

## Authors’ contributions

Both authors contributed to the development and writing of this manuscript and approve the final content.

## Authors’ information

JS is Research Associate in the Philosophy Department and Graduate School of Education of the University at Buffalo, NY, USA; JG is Chief of the Neuroethics Studies Program, Pellegrino Center for Clinical Bioethics, Georgetown University Medical Center, Washington, DC, 20057, USA and Clark Faculty Fellow, Human Science Center of the Ludwig-Maximilians Universität, Munich, Germany.
